# A mixed-method study exploring barriers and facilitators to midwives’ mental health in Ontario

**DOI:** 10.1186/s12905-023-02309-z

**Published:** 2023-04-01

**Authors:** Elizabeth K. Darling, Lindsay N. Grenier, Rachel K. MacKenzie, Claire Ramlogan-Salanga, Elizabeth C. Cates, Riley Graybrook, Karline Wilson-Mitchell

**Affiliations:** 1 McMaster Midwifery Research Centre (MMRC), Department of Obstetrics & Gynecology, McMaster University, Hamilton, ON Canada; 2 Midwifery Education Program, Faculty of Community Services, Toronto Metropolitan University, Toronto, ON Canada

**Keywords:** Mental health, Midwives, Workplace stress, Burnout, Model of care, Ontario midwives

## Abstract

**Background:**

There is a paucity of information regarding the mental health of midwives working in Ontario, Canada. Many studies have investigated midwives’ mental health around the world, but little is known about how the model of midwifery care in Ontario contributes to or negatively impacts midwives’ mental health. The aim of the study was to gain a deeper understanding of factors that contribute to and negatively impact Ontario midwives’ mental health.

**Methods:**

We employed a mixed-methods, sequential, exploratory design, which utilized focus groups and individual interviews, followed by an online survey. All midwives in Ontario who had actively practiced within the previous 15 months were eligible to participate.

**Findings:**

We conducted 6 focus groups and 3 individual interviews, with 24 midwives, and 275 midwives subsequently completed the online survey. We identified four broad factors that impacted midwives’ mental health: (1) the nature of midwifery work, (2) the remuneration model, (3) the culture of the profession, and (4) external factors.

**Discussion:**

Based on our findings and the existing literature, we have five broad recommendations for improving Ontario midwives’ mental health: (1) provide a variety of work options for midwives; (2) address the impacts of trauma on midwives; (3) make mental health services tailored for midwives accessible; (4) support healthy midwife-to-midwife relationships; and (5) support improved respect and understanding of midwifery.

**Conclusion:**

As one of the first comprehensive investigations into midwives’ mental health in Ontario, this study highlights factors that contribute negatively to midwives’ mental health and offers recommendations for how midwives’ mental health can be improved systemically.

## Background

Midwives face a number of occupational risks to mental health, including working long, unpredictable hours involving nights and other unsociable hours, and experiences of workplace abuse from a variety of sources, such as patients, patients’ relatives, colleagues, or hospital administration [1]. Burnout and stress, which are strongly associated with mental health issues including depression and anxiety, are common among midwives [2–4]. A recent meta-analysis suggests that organizational factors associated with higher levels of burnout in midwives include a lack of staff and resources, low salaries, poor professional recognition and organization, and a negative work environment [5]. Midwives are also at serious risk of post-traumatic stress disorder (PTSD) as they may be exposed to traumatic events such as the death of a baby or mother, emergencies at births, and experiencing or witnessing violence [6–8].

While there has been research into the levels of stress and burnout experienced by midwives around the world, little is known about the mental health of Ontario midwives who work predominantly in midwifery-led continuity of care models [3–5]. Many facilitators of mental health, such as routines and workload management, are incompatible with the current work arrangements of midwives in Ontario. The objective of this study was to gain a deeper understanding of the factors contributing to the mental health of Ontario midwives, with the long-term goal of using these findings to identify interventions that would effectively support good mental health for midwives.

### The Ontario midwifery context

Midwifery in Ontario, Canada was formally recognized as a profession and publicly funded in 1994 [9]. By 2020, midwives cared for roughly 1 in 5 pregnant women and people giving birth in the province [10]. The philosophy of midwifery care in Ontario centres the unique needs, values, and preferences of pregnant women and people, who are referred to as ‘clients’ as a reflection of this philosophy [11]. Ontario midwives work primarily in community-based midwifery-led continuity of care practice groups [12], hold privileges at hospitals, and offer their clients a choice of giving birth at home or in hospital, or, where available, at a birthing centre [13]. Midwifery practice groups are partnerships or small businesses, and midwives who are not practice partners/owners are independent contractors to the practice group [12]. Midwives are paid per client after completion of a full course of antenatal, intrapartum, and postpartum care, with funding flowing through their practice group [12]. Approval of practice caseload and new midwifery practice groups is managed by the Ontario Ministry of Health [14]. Since 2018, a very small portion of Ontario midwives have worked in salary models that facilitate work that is not compatible with course of care funding [15]. Also in 2018, the Human Right’s Tribunal of Ontario (HRTO) determined that midwives’ compensation has been subject to gender discrimination [16]. In 2022, the Ontario government withdrew their appeal of the HRTO decision and the finding of the tribunal was accepted [17].

## Methods

We employed a mixed-methods, sequential, exploratory design for this study. In Phase I, we conducted focus groups and individual interviews to gather qualitative data. We then used the findings from Phase I, along with existing literature, to develop Phase II - an online survey to gather quantitative data to further explore and corroborate the qualitative findings.

### Participant recruitment

Our intent was to broadly examine the topic of mental health and identify factors that promoted good mental health, as well as examine factors that contributed to mental illness. For this reason, at all phases of the research we made an open call for participation to all midwives in Ontario who had practiced midwifery within the past 15 months and did not limit participation to people who had been diagnosed with mental illness. We recruited Phase I participants from across the province via an open call in the weekly Association of Ontario Midwives (AOM) email Memo, via social media platforms, and by directly emailing midwifery practice groups. This was followed by snowball sampling and targeted sampling to recruit a diverse group of midwives (e.g., with respect to geography, race, age, gender, sexual orientation, etc.) from a variety of settings across the province, with varied client demographics and mental health experiences. Recruitment for Phase II was via the AOM’s email Memo, social media, and direct emails to midwifery practice groups. Ethics approval was obtained from the Hamilton Integrated Research Ethics Board (HiREB; project #12,590 & #10,995).

### Data collection

Three members of our research team conducted semi-structured individual and focus group interviews using interview guides with open-ended questions to explore participants’ views and experiences pertaining to facilitators and barriers to mental health within the midwifery profession. We offered participants who identified as racialized the option to participate in an individual or focus group interview that only included racialized participants and was led by a racialized member of the research team. The interview guides were informed by the literature on mental health in midwifery. Participants provided informed consent and completed an online demographic questionnaire prior to the interviews. Interviews were conducted via Zoom and were audio recorded.

The Phase II online survey was developed based on the themes identified in Phase I and was piloted and revised based on feedback from midwives prior to being conducted. We also used several previously developed measures, including Traumatic Events in Perinatal Care List [7], the Copenhagen Burnout Inventory [18], the Depression, Anxiety and Stress Scale (DASS-21) [4, 19], and Perceptions of Empowerment in Midwifery – Modified [2, 20, 21]. Questions regarding bullying in the workplace were adapted from material published by the Association of Ontario Midwives [22]. The surveys were collected using REDCap, a secure web-based platform. Consent was obtained electronically when participants accessed the survey online. Our target sample size was 274, based on there being 949 Registered Midwives in Ontario at the time we designed the study, to support a margin of error of 5%, and a confidence level of 95 [23]. This represented a target response rate of 28.6%.

### Data analysis

We approached Phase I data analysis using a constructivist perspective [24]. The interviews were professionally transcribed and deidentified. Two researchers analysed the transcripts using NVivo software and following Sandelowski’s method of qualitative descriptive analysis [25]. Analysis was staged, beginning with open-coding to summarize and describe the data, followed by focused coding to identify and categorize themes. We used an iterative approach to data collection and analysis, in which findings in the initial interviews informed refinement of the interview guides to allow us to explore themes that arose from the data. The researchers who conducted the coding wrote memos describing preliminary themes. The memos were reviewed and revised by the study team, referring to the data, until consensus was reached on final themes. To ensure trustworthiness of the analysis, we conducted member checking through meetings with two groups of midwives (a total of approximately 20 midwives, some of whom participated in Phase 1) from across the province for feedback on the preliminary findings, to check our findings for accuracy and resonance with their experiences.

Phase II survey data were analyzed using STATA software. For this manuscript, we conducted descriptive statistical data analysis only.

## Results

In Phase I, we conducted 6 focus groups and 3 individual interviews with 24 participants between August 4, 2020 and October 30, 2020. One participant participated in both a focus group and individual interview. The Phase II online survey was open between February 5, and April 14, 2021, and had 275 respondents, exceeding our target sample size of 274. For clarity, we refer to midwives who took part in Phase I as participants and midwives who to took part in Phase II as respondents. The characteristics of the Phase I participants and Phase II respondents are shown in Table 1.

**Table 1:** Focus Group & Interview Demographic Characteristics

Characteristic	Focus Groups & Interviews n (%)	Survey n (%)
Age	**(n = 24)**	**(n = 263)**
20–29	2 (8.3)	37 (14.1)
30–39	5 (20.8)	89 (33.8)
40–49	6 (25.0)	97 (36.9)
50–59	6 (25.0)	35 (13.3)
60–69	1 (4.2)	5 (1.9)
Did not wish to answer	4 (16.7)	0 (0)
Born in Canada	**(n = 24)**	**(n = 268)**
Yes	19 (79.2)	232 (86.6)
No	5 (20.8)	36 (13.4)
Identifies as Racialized	**(n = 24)**	**(n = 268)**
Yes	9 (37.5)	29 (10.8)
No	15 (62.5)	235 (87.7)
Did not wish to answer	0 (0)	4 (1.5)
Person with disability	**(n = 24)**	**(n = 268)**
Yes	6 (25.0)	41 (15.6)
No	18 (75.0)	222 (84.4)
Did not wish to answer	0 (0)	5 (1.9)
Has ever taken a medical leave for mental health reasons	**(n = 24)**	**(n = 267)**
Yes	3 (12.5)	38 (14.2)
No	20 (83.3)	227 (85.0)
Did not wish to answer	1 (4.2)	2 (0.8)
Number of years practicing midwifery in Ontario [combined].	**(n = 24)**	**(n = 256)**
< 1 (currently in first year of practice)	0 (0)	20 (7.8)
1–5	8 (33.3)	85 (33.2)
6–10	4 (16.7)	62 (24.2)
11–15	5 (20.8)	53 (20.7)
16+	5 (20.8)	36 (14.0)
Did not wish to answer/Did not respond	2 (8.3)	0 (0)

Phase I participants primarily worked as practice associates (46%) in a group practice arrangement (79%) working full time (63%). Most participants had a career prior to becoming a midwife (63%) and had entered midwifery through the Ontario undergraduate midwifery education program (83%). Phase II respondents primarily worked as practice partners (44%) in a group practice arrangement (89%) working full time (77%). Most respondents worked in a shared call model (74%), with between 11 and 15 midwives working at the practice (40%) and had entered the profession through the Ontario midwifery education program (87%). Compared to Phase I participants, survey respondents were more likely to be born in Canada (87% vs. 79%), identify as white (88% vs. 71%), and not work in a rural or remote area (75% vs. 67%). Phase II respondents were less likely to identify as heterosexual (67% vs. 75%) or racialized (11% vs. 38%), or report having a chronic illness or disability (16% vs. 25%).

**Fig. 1:**
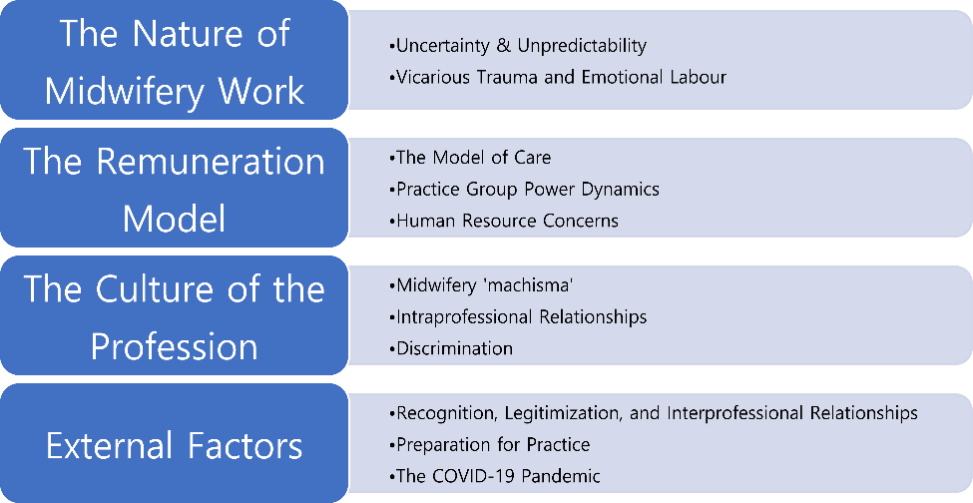
Thematic findings impacting midwives’ mental health

Our qualitative analysis identified four broad factors that impact midwives’ mental health: the nature of midwifery work, the remuneration model, the culture of the profession, and external factors. Within each of these broad categories, we identified additional sub-themes (see Fig. 1).

Our qualitative findings were corroborated through our quantitative analysis of survey respondents’ ratings regarding how numerous factors impacted their mental health (see Table 2). The survey results provided insight into the broad range of experiences across the profession, including differences between midwives in how they were impacted by the factors we explored. Both the qualitative and quantitative findings related to the themes and sub-themes are described below. We present findings from Phase II regarding proposed solutions in Table 3.

**Table 2:** Factors impacting mental health (from most to least Negative/Very Negative ratings)

Factor	n	mean (SD)	% Negative or Very Negative	% Neutral	% Positive or Very Positive
Other health professionals’ lack of understanding of midwifery (e.g., lack of understanding re: role, scope of practice, training)	275	1.78 (0.7)	89.1%	9.1%	1.8%
The physical impact of stress resulting from midwifery	274	1.90 (0.79)	81.8%	15.3%	2.9%
Ability to get adequate sleep	275	1.88 (0.97)	81.1%	10.2%	8.7%
Midwifery’s work-life balance	275	1.88 (0.9)	80.4%	12.4%	7.3%
Ability to maintain healthy eating habits	274	1.99 (0.96)	79.6%	10.6%	9.9%
Comfort with level of preparation upon graduation for running a business	274	1.98 (0.77)	74.8%	23.4%	1.8%
Time available for exercise	274	2.06 (0.98)	74.5%	15.7%	9.9%
Midwifery’s on-call schedule	274	2.23 (0.94)	66.1%	23.7%	10.2%
Power dynamics within midwifery practice groups	272	2.12 (0.99)	64.3%	27.2%	8.5%
Ability to maintain social relationships outside of work	274	2.35 (1.04)	61.7%	22.6%	15.7%
Flexibility of the midwifery model of care	275	2.32 (1.1)	61.5%	19.3%	19.3%
Level of preparation upon graduation for the nonclinical aspects of the midwifery profession (e.g., business, interpersonal, etc.)	275	2.39 (0.82)	58.9%	33.1%	8.0%
Availability of time off/support to deal with illness	275	2.34 (1.14)	58.2%	22.6%	19.3%
Level of compensation for work performed	275	2.55 (1.03)	56.7%	21.5%	21.8%
Lack of accountability of midwifery practice groups/practice partners	274	2.26 (0.99)	54.7%	38.3%	6.9%
Bullying within midwifery practice groups	273	2.27 (0.96)	54.6%	38.5%	7.0%
Availability of time off/support following difficult or traumatic events	273	2.52 (1.18)	52.4%	21.6%	26.0%
Level of mental health support at work	274	2.48 (0.96)	51.1%	34.7%	14.2%
Payment structure of the midwifery profession	275	2.68 (1.06)	48.0%	26.2%	25.8%
Ability to maintain relationships at home	275	2.66 (1.03)	47.6%	29.5%	22.9%
Difficulty filling positions/retention within midwifery practice groups	275	2.51 (0.81)	44.0%	49.5%	6.6%
Expectations from clients	274	2.69 (0.84)	43.8%	40.2%	16.1%
Physical nature of midwifery work	275	2.73 (0.95)	41.1%	37.1%	21.8%
My level of decision-making power/autonomy within my midwifery practice group	273	2.96 (1.2)	38.1%	22.3%	39.6%
Opportunity for time off	275	2.97 (1.16)	37.1%	22.2%	40.7%
Differing philosophies within midwifery practice groups	275	2.68 (0.81)	36.7%	51.3%	12.0%
Relationships with consultants	275	2.97 (1.03)	35.3%	27.6%	37.1%
Clients’ lack of understanding of midwifery (e.g., lack of understanding re: role, scope of practice, training)	275	2.70 (0.68)	35.3%	57.5%	7.3%
Relationships with hospital nursing staff	273	3.03 (1.08)	35.2%	22.3%	42.5%
Job security in the midwifery profession	274	3.13 (1.21)	34.3%	17.9%	47.8%
My level of agency regarding my career path/goals	274	3.00 (1.07)	33.6%	30.7%	35.8%
Relationships with hospital administration	275	2.92 (0.98)	33.1%	34.9%	32.0%
Requirements to work to the full scope of practice (e.g., epidurals, induction of labour)	275	2.91 (0.91)	30.6%	46.2%	23.3%
Transitioning from a student to a new registrant position	275	3.14 (0.98)	30.2%	29.1%	40.7%
My level of agency within my practice group	272	3.08 (1.13)	29.8%	27.2%	43.0%
Ease of obtaining hospital privileges	273	2.94 (1.02)	28.9%	42.5%	28.6%
Restrictions on ability to work to the full scope of practice (e.g., epidurals, induction of labour)	273	2.82 (0.82)	27.5%	58.2%	14.3%
Broadening of the ways in which midwives can work (e.g., Expanded Midwifery Care Models)	274	3.16 (1.04)	22.6%	37.6%	39.8%
Level of support at home	275	3.66 (1.07)	18.2%	15.6%	66.2%
Increased utilization of delegation from other healthcare providers (e.g., medical directives from physicians to prescribe substances not in the midwife’s pharmacopeia)	275	3.20 (0.86)	16.4%	47.6%	36.0%

**Table 3:** Midwives’ ratings of the potential helpfulness of proposed solutions to support mental health

Potential Solution	n	Mean (SD)	% Not helpful at all	% Slightly or Somewhat Helpful	% Very or Extremely Helpful
More access to alternative working models for midwives	275	4.37 (0.90)	2%	14%	85%
Protected time off following traumatic events	273	4.35 (0.84)	1%	13%	87%
Support for part-time work	275	4.34 (0.93)	3%	11%	87%
Structured support following traumatic events	273	4.3 (0.85)	0%	16%	84%
Increased compensation for work	273	4.16 (0.96)	1%	24%	75%
More protected off-call time	274	4.1 (1.10)	3%	22%	75%
Increased access to a pool of mental health professionals familiar with the challenges unique to midwifery	272	3.96 (1.12)	4%	24%	72%
Professional business management services MPGs	271	3.94 (0.99)	3%	26%	72%
Increased insurance coverage/affordability of mental health services	272	3.81 (1.20)	7%	27%	67%
Increased resources for administrative support	273	3.78 (1.06)	4%	30%	66%
Increased benefits for individual counselling	271	3.7 (1.24)	7%	30%	63%
Professional conflict mediation services for midwifery practice groups	272	3.63 (1.23)	7%	35%	58%
More training or support about the business aspects of midwifery work	274	3.63 (1.10)	5%	34%	61%
More support for new registrants transitioning to clinical practice	274	3.61 (1.10)	6%	35%	60%
Supportive mentorship from an experienced midwife outside of your practice	271	3.43 (1.25)	9%	41%	50%
Increased benefits for services such as massage, chiropractic care, osteopathy, acupuncture, etc.	273	3.37 (1.36)	12%	40%	48%

### The nature of midwifery work

Within the theme of *The Nature of Midwifery Work*, we identified two subthemes, *Uncertainty and Unpredictability*, and *Vicarious Trauma and Emotional Labour*, which were related to the ways in which midwifery work negatively impacts mental health. Overall, while many participants described midwifery work as deeply rewarding and work that they love, there was wide variation in how they found the work to energize or drain them. Less than half of respondents felt energized by their work as a midwife (45%) or felt their expectations matched the realities of working as a midwife (48%).

### Uncertainty and unpredictability

For some participants, the uncertainty and unpredictability related to long periods of being on-call had a significant negative impact on their sense of mental wellbeing. The majority of survey respondents felt that midwifery’s work-life balance (80%), on-call schedule (66%), and (in)ability to get adequate sleep (81%) had a negative impact on their mental health. Participants spoke extensively about the impact that being on-call had on their anxiety levels, and many felt like they were never able to be truly ‘off’ and relax or unwind. Thoughts about midwifery permeated participants’ off-call lives.

*[Y]ou can’t probably sleep deep enough, because you have to be constantly aware of your cellphone…my first year…I couldn’t be away from my phone. I was totally scared to be out of the house because I was thinking about the signal of my phone, how far I was going, and I’d even see if there was so much traffic…just the idea of being ready all the time…I was so panicked [that] I was going to miss the calls. (Focus Group 1, Participant 2)*

While the discussion of uncertainty and unpredictability often related to being on-call, for some it extended beyond scheduling to include uncertainties as to what situations work might entail each day.

*There is almost no certainty…My call day could be anything. I think it does take a certain mindset…about accepting uncertainty, being willing to remain in the uncertainty, and that’s not easy. (Focus Group 3, Participant 7)*

Participants described how unpredictable scheduling can make it harder to access mental health supports, because midwives’ call work inhibits them from committing to things like a weekly exercise class or support group, despite knowing that these activities would have a positive effect on their mental health.

### Vicarious trauma and emotional labour

Participants spoke about two broad ways in which the emotional demands of midwifery work negatively impact their mental health, namely their experiences attending traumatic births and the general emotional labour involved in caring for their clients. Traumatic birth experiences greatly impacted midwives’ mental health as they would relive distressing situations over and over in their minds. As one participant described,

*I know so few midwives who I graduated with who are still practicing six years later, because it’s almost like the career has pushed them out. They’re exhausted. They’ve had too many stillbirths. They’ve had not enough support…when you are off call people are still calling you and texting you, and saying, ‘I know you’re off call, but…’ How do you get time to heal yourself when you have had a traumatic outcome or traumatic birth, and especially when we do get close to our clients? It’s too much, and it does accumulate, and I found myself having headaches, and not being able to sleep, and not being able to unwind, and not being able to eat properly. Your body’s telling you something. (Focus Group 2, Participant 3)*

The trauma experienced by midwives attending births (as measured by the Traumatic Events in Perinatal Care List) included interpersonal disrespect (83%) such as witnessing a client’s dignity being ignored or her/their wishes overridden, or experiencing fear of or actual death/injury of a client or baby (77% and 76% respectively), and witnessing, performing, or participating in a procedure that was not in the client’s and/or the baby’s best interest (71%). Half of the respondents had experienced abusive care or management and only 3% had never experienced any of the traumatic events described above.

*There’s so much trauma, man. I feel like there’s a lot of midwives that are understanding and accepting and compassionate about other people’s trauma, our client’s trauma, sexual trauma, all that other stuff. We take in a lot of trauma ourselves, and we have no way of working [it] out. Nobody understands. (Interview 3)*

While most participants had experienced traumatic births as part of their work as a midwife, those who felt most negatively impacted by this often reported feeling that there was not adequate time to recuperate after traumatic experiences and/or that there was inadequate support and understanding within their midwifery practice group. Lack of support or time off following traumatic events was also identified as negatively impacting mental health by a majority of survey respondents (52%). Participants also reported a lack of understanding from friends and family about what they had experienced.

Aside from traumatic experiences, some participants also generally described the nature of birth work as emotionally exhausting, especially when coupled with external factors that increased stress. While the survey responses indicate that the vast majority of midwives believe that the work they do is valued by clients (97%), client expectations or lack of understanding had a negative impact for a notable portion of respondents (44%). One participant described how this created unrealistic emotional demands:

*It sometimes feels like a lot of emotional bandwidth [is expected by clients] that I don’t think is a realistic expectation, and it’s certainly not an expectation [for] other healthcare providers…I think some clients inappropriately use us as a Google search in terms of inappropriate pages. I think some clients feel like they’re getting a friend. Very inappropriate expectations out of a healthcare provider when you look at what other clients/patients expect from similar obstetric providers, whether it’s doctors - like OBs or family doctors. (Focus Group 2, Participant 5)*

Participants reported that client relationships impacted their mental health, and some described clients’ expectations for emotional support to be emotionally exhausting and felt like this emotional labour wasn’t valued.

## The remuneration model

Within the theme of *The Remuneration Model*, the following sub-themes were identified: *The Model of Care*, *Practice Group Power Dynamics*, and *Human Resource Concerns*. Participants identified that the current funding arrangements for Ontario midwives can have a detrimental impact on midwives’ mental health. Midwives also spoke about their work being undervalued and underpaid, particularly, although not exclusively, with regards to the emotional labour involved in midwifery work. A notable proportion of survey respondents agreed that the level of compensation for work performed (57%) and the payment structure of the profession (48%) had negative impacts on their mental health.

### The model of care

Many participants identified that the model of midwifery care in Ontario, which requires midwives to provide on-call intrapartum care, made it difficult for some midwives to maintain good mental health and find a desirable work-life balance, particularly at certain life stages. Some stated that they were unsure how sustainable the profession would be for them long-term as they started a family or got older. Many participants mentioned the high attrition rate of midwives and spoke to how rigidity in the model contributes to midwives leaving the profession because they are unable to find work arrangements that are personally viable.

*You’re a registered midwife, so you must do clinic, you must do births, you must do postpartum. You have to do all of it or nothing. There are no options for saying, you know what? I’m really, really super great at the postpartum aspect of it. You guys do the delivery. I’ll take over postpartum care, and still get paid for it as a midwife, and still maintain and retain my RM status. I think that a lot of midwives who might want to do something else under our scope, but…[they] are afraid of losing their designation, and I don’t think that’s fair. When doctors specialize, or reduce, or whatever…they don’t stop being called Dr. So and so. Why do we have to stop being called registered midwives or lose our designation at all? (Focus Group 2, Participant 3)*

Having a variety of work options was seen as key for improving midwifery retention, and Phase I participants believed that more funding of Expanded Midwifery Care Model (EMCM) or alternative ways of working would help support work-life balance, and therefore improve mental health. This was corroborated by the survey, in which 40% of respondents agreed that broadening the ways in which midwives can work, such as expanded models, had a positive impact on their mental health, while the lack of flexibility of the model had negative impacts (62%). We found variation in preferences, with some participants mentioning how much they appreciated and valued the autonomy of midwifery-led continuity models, while others who worked in expanded or alternative models spoke about the benefits of being an employee and pointed out that it can be possible to retain autonomy in employee models.

### Practice group power dynamics

Participants described how power dynamics that arise as a result of funding for midwives flowing through midwifery practice groups and the way in which practice groups operate impacts their mental health. Some midwives spoke about feeling powerless within practice groups and finding it challenging to advocate for themselves or raise concerns due to a lack of power relative to senior midwives or practice partners. In some practice groups, non-partner midwives experienced a lack of transparency from practice partners and felt they were not fully informed about what was happening at the practice. Newly registered midwives and students felt particularly vulnerable to power imbalances. Negative power dynamics left midwives feeling unsupported by their practice groups, which had negative impacts on mental health. Given the approval requirements for funding new midwifery practice groups, the first midwives to establish a practice group in a community effectively end up having a monopoly on midwifery services in that area, and the only option for newer midwives who wish to work in that location is to join the existing practice group. The lack of alternative work options sustains power imbalances because newer midwives tolerate abuses of power in order to maintain the ability to work in the community of their choice.

*So much of this issue…is about power. This is a recurring theme. Ultimately though, the funding model gives the power, generally speaking, to the partners in a practice, who…can be as arbitrary or not as they like… I think until we actually address the power structure, which let’s face it, is money driven at the very end of the day, we’re going to face these challenges. (Focus Group 3, Participant 7)*

Participants also described how unequal power dynamics sometimes contributed to bullying and harassment within practices, which negatively impacted midwives’ mental health. Bullying behaviours were often perpetrated by midwives who were ‘paying it forward’ based on their own challenging experiences as students and/or new registrants. This was supported by survey findings, which indicated 64% agreed that midwives’ mental health is negatively impacted by power dynamics in practice groups.

In contrast, 65% of respondents reported feeling involved in the decision-making process for important decisions within the practice, and participants who experienced supportive and healthy practice group dynamics reported that this very positively impacted their mental health. For example, participants spoke about the benefits of having practice partners who were willing to accommodate different practice arrangements, such as part time work, especially for midwives who were older or who had different health needs, and how this level of support was very beneficial.

### Human resource concerns

The midwifery practice group-based funding arrangements create a number of human resource (HR) concerns, which were corroborated by survey findings. Participants spoke about how non-partner midwives often function similarly to employees, but they do not have the same benefits and protections that true employee have. Participants described challenges with having other midwives act in an HR role within a practice group, as they felt uncomfortable raising issues to this person who might end up supporting them at a birth or vice versa. These conflicts of interest arose because of the dual roles played by practice partners.

*I think the hard part is…if you are going to have somebody that is [responsible for] HR in your practice, it’s probably somebody…you work closely with and if perhaps that’s somebody you don’t get along with as well or that you have issues with, it doesn’t make things easy…So, I think having a third-party person or service that you can call on and bring into the practice, I think goes along with that accountability side of that. (Interview 1)*

Participants felt it was important to have specific and dedicated HR personnel within clinics to avoid conflicts of interest that might arise, for example, when trying to navigate or resolve issues of bullying and harassment. Many participants also spoke about the potential benefits of having an outside party in terms of accountability and to help ensure changes actually happened or were implemented within practices.

*I think [the] [midwifery practice group]…structure, it lends itself to a lot of dysfunction…I [work in an employee model program]…there’s some bureaucracy that I have to navigate…the flip side of that is I have an HR person. I have an IT person. I have a finance person…administrators who work in the supporting aspect. They have a manager. I have…clinical assistants. They have a manager. All of those people create a system that can’t malfunction in the same way, because there are too many people watching. (Focus Group 5, Participant 20)*

Concerns around a lack of accountability of practice groups and partners negatively impacted 55% of respondents.

Another key HR issue for midwives relates to time off work. Midwifery practice groups do not always have established policies regarding time off for illness or extraordinary circumstances. Many midwives spoke about the barriers to taking time off, because there was either little support from their practices to do so (for example, after a stillbirth), or because they felt guilty when they did take time off. Midwives were often very aware that if they took time off it would mean added work and stress for their co-workers, and this made them reluctant to do so. These circumstances result in midwives delaying or avoiding taking time off when they are experiencing mental health concerns to not be a burden to their colleagues.

## The culture of the profession

The culture of the midwifery profession was also seen to have a large impact on the mental health of midwives. Within the theme *The Culture of the Profession*, the following sub-themes were identified: *Midwifery ‘Machisma’*, *Intra-professional Relationships*, and *Discrimination*. While participants reported that positive aspects of the culture of the profession, such as midwives’ passion for and belief in midwifery, support them to navigate the challenging parts of their work, they also described how each of the three sub-themes could drive midwives to feel that they needed to take an extensive break from the profession, despite their love of the work itself.

### Midwifery ‘Machisma’

Many participants spoke about the culture of midwifery as being one in which people just had to ‘push through’ and ignore their needs, often at the expense of their mental and physical health. Participants reported issues with midwifery culture, including lack of time off and support. Two-thirds (63%) of survey respondents felt they could not take time off from work when needed and 58% reported that the (in)ability to take time off and receive support to deal with illness had a negative impact on their mental health. Participants said that expectations to push through came from both themselves and from other midwives, and entailed addressing their own needs last after the needs of clients and other professionals:

*[P]art of being a midwife or at least part of the way I was taught to be a midwife, taught you to push aside your own needs. Your sleep, your hunger, your need to go to the bathroom, as well as put aside the emotional things in your personal life and the things that you carry with you, if your kid is at home sick, you keep working. If you have a fight with your partner and your pager goes off, you go to the birth. I think that a lot of health care workers have this, but I also feel there is…midwifery martyrdom, where the client comes first, that’s the priority, always…never take time off. Don’t worry about your own needs and then you are a good midwife. (Focus Group 4, Participant 15)*

Participants spoke about their work ethic creating an unrelenting pressure and feeling that they should always be on or working at their full capacity. Some participants mentioned that working in alternative models of practice, or requiring a leave, was seen within the profession as lesser, or that other midwives did not consider them a ‘real’ midwife, and it was hard to get respect from other midwives if they chose to work in an alternative way. Participants also spoke about experiencing pressure to push through after a loss at work which exacerbated the trauma they had experienced.

*[I]t’s important to acknowledge a little bit of a culture of machisma in midwifery, of feeling proud of being tough enough to keep going. I don’t know what that’s about, but I think we can’t underestimate the impact that it has on how people see themselves, how people feel like they have to behave, and how people see other people, and how willing they are to talk about what they might need. (Focus Group 3, Participant 8)*

As mentioned in the sub-theme *Vicarious Trauma and Emotional Labour*, lack of adequate time off to process and recover from traumatic experiences exacerbated the impact of traumatic events for participants. While some participants shared that their midwifery practice group agreed to prioritize time off after traumatic events, this was not a common practice amongst the focus group participants. Finally, participants found it extremely challenging to take time off of work because of the feelings of guilt, as described earlier. Midwives felt they were a burden on their co-workers if they took time off, even if they were sick or needed to attend to medical needs. These feelings of guilt and pressure were further perpetuated and exacerbated by a midwifery culture that suggests that midwives ‘push through’, even to the detriment of their own health.

### Intra-professional relationships

While majority of survey respondents reported positive, supportive relationships with other midwives, with 87% reporting they have the support and backing of their fellow midwives, this finding was accompanied by widespread reports of negative interprofessional interactions. When negative intra-professional relationships occurred, they had a significant negative impact on midwives’ mental health. Participants identified that there were limited resources in midwifery, which sometimes resulted in midwives being mean to each other and overly competitive, even pulling each other down in an effort to get ahead, like ‘crabs in a bucket.’ Participants also described tension between colleagues who had different philosophies about midwifery, either in terms of client care, or with regards to challenging hospital status quo. Some participants experienced interpersonal challenges when other midwives failed to recognize midwifery colleagues’ mental health issues. There was extensive discussion of intra-professional bullying in the focus groups, which was echoed in the survey. 98% of respondents reported experiencing some form of bullying, 44% experiencing some form of bullying regularly, and over half reporting that bullying within practice groups negatively impacted their mental health (55%). Respondents reported experiencing excessive or unjustified criticism often (21%), and unrealistic goals or deadlines that resulted in being overloaded with work (20%). Participants described their experiences of bullying, and how these led to consequences that included intense anxiety and fear around attending practice group meetings, having to quit their workplace, and overall poor mental health.

*Why are we so mean to each other? I see so many midwives who are just out to get their colleagues, out to get the other [midwifery practice groups].…we’re better than you, blah, blah, blah. Why? It’s almost like we’ve been given this little tiny piece of the medical world, and we’re going to claw and scratch our way to be at the top of that, no matter who you step on, and unfortunately, we’re stepping on each other, as opposed to rising all of us up so that midwifery itself is elevated to the respect and the status that it should be, we’re stepping on each other. (Focus Group 2, Participant 3)*

Some participants talked about individuals being targeted, and how power structures allowed bullying to continue or escalate without any repercussions. They described a lack of accountability when bullying happened and noted that there were few protections in place to ensure that it was adequately dealt with. These issues also fed into the human resource challenges related to conflicts of interest described previously under Human Resource Concerns.

*[F]or myself and my fellow associates [the bullying] was psychological, like making fun of us in front of clients…say for instance, being more conservative as an NR [new registrant] than the midwives and saying, ‘Oh should we consult?’ and then being kind of ridiculed in front of your clients and consultants, that kind of stuff. But also, I’ve been physically assaulted by one of the midwives as well…it just kind of ran the gamut. (Focus Group 5, Participant 19)*

These very negative experiences were not shared by most respondents, yet despite the majority reporting good relationships with their midwife colleagues (87%), 90% did not feel adequately prepared for the interpersonal aspects of the profession.

### Discrimination

Finally, participants described experiencing discrimination at work, including racism, ageism, and homophobia. These experiences of discrimination made them feel othered within practice groups, and occasionally worried about their safety or experiences with clients. For some racialized midwives, the stress of racism compounded the effects of an already stressful occupation. As one participant described,

*There is a lot of stress at work because we are midwives, but then you add on top of that, the fact that we are racialized midwives who are constantly being disrespected at the hospital or being bullied in our own clinics or seeing evidence of our clients who are racialized being bullied and disrespected and discriminated against and often … we can’t say anything because we are the only one in the room and the consequences of saying something are going to come back to you. So we hold it and we hold it and we hold it, and for me I would hold and my back would go out and I would be out for three days. (Focus Group 6, Participant 3)*

Racism was identified as a factor that both shaped the culture of the profession and influenced external factors that are discussed under the next theme below. Racialized participants identified gaps in policy and organizational structures to address racism, which resulted in the onus to address racism falling on individual racialized midwives.

Participants also raised concerns about ageism. Some participants spoke about this in terms of feeling less trusted as a younger midwife, while others spoke about how they felt undervalued by and less connected to new, younger midwives in their practice group. Participants also described the challenges of aging within the profession in terms of systemic issues that made work unsustainable, and interpersonal issues such as philosophical differences between different generations of colleagues.

A few participants spoke about homophobia and the challenges being a queer midwife. Often this discrimination occurred in the form of microaggressions rather than overt offensive comments. In particular, queer midwives described discomfort when other midwives discussed their families without recognizing that this might lead to unwanted questioning of the queer midwife about their family. Participants pointed out how the common practice of midwives sharing a description of their hobbies, interests, and families on practice group websites can impact queer midwives’ safety. Participants also spoke about experiencing discrimination from clients or worrying about how clients were perceiving them. For example, one queer midwife spoke about fears that a family would learn that they were queer.

*I wouldn’t say that I experienced homophobia necessarily, certainly not…outright bigotry, but I worked for two years in a suburban community…that’s quite conservative, and heteronormativity was exhausting. After two years there, I was exhausted…it came from everywhere. It came from clients. It came from all of the nurses on the labour floor, all OBs, and a lot of the midwives…it was just a complete invisibility of queer identities and an idea that anyone might not be married to a man and have children… Being put in positions where I had to decide to out myself or lie…at first, it didn’t seem like that big a deal, or it’s just is the way it is, but after a couple of years, I couldn’t have taken much more of it…There’s a fatigue in all of that. (Focus Group 5, Participant 20)*

## External factors

Participants identified several factors external to the midwifery profession or clinical work that also impacted midwives’ mental health. Within the theme of *External Factors*, we identified the following sub-themes: *Recognition, Legitimization, and Interprofessional Relationships*; *Preparation for Practice*; and *The COVID-19 Pandemic*.

### Recognition, legitimization, and interprofessional relationships

Participants spoke extensively about challenges related to the recognition and legitimization of midwifery, which were closely tied to challenges they discussed with interprofessional relationships. Midwives described having to fight hard to be recognized for their work, their education, and their ability to provide care to pregnant people—both with other health professionals, but also with friends, family, and the general public. Generally, participants felt that there was a lack of understanding of their experiences, their education, and their training.

*I guess perception or lack of understanding of what we do in the general public, and that includes in my friendships, in my family life…I mean, I’m going and doing a manual removal and [its] just [a] horrifying situation…and then I’m talking to my friend on the phone, and she’s like, ‘Oh, but you’re a doula, so it can’t be that bad’…Okay, I’m just going to go die in the tub now. I’m just going to go cry myself to sleep. I just kind of feel like my identity is so misunderstood over, and over, and over, and I feel like that again is retraumatizing…I think if you’re a doctor and you say, ‘I had a really hard shift,’ people will be, ‘Wow, that must be so hard. You do all this amazing work’…there’s this support. (Focus Group 2, Participant 6)*

Participants felt their profession was not well understood by some clients, including their role, scope of practice, and training, and over a third of respondents (35%) reported that this negatively impacted their mental health. One participant commented that midwifery being marketed as a ‘free service’ (since it is covered by public health insurance and available to uninsured residents) can also contribute to midwifery being devalued by the general public. Participants also noted that online practice group profiles, particularly the language and branding which midwives use when they present themselves to the public, often downplays midwives’ credentials in a way that might serve to delegitimize their professional expertise and facilitate clients’ transgression of professional boundaries (e.g., asking invasive personal questions).

*If you look up any doctor, any other provider …where there’s a bio on their website, it says where they went to school, it says where they have worked, and it says what their area of interest is if they do research. We have these…totally unprofessional bios, and that’s the standard in the profession. It’s expected that you talk about what you like to do in your spare time, and your cute kids, and your husband, and whether or not you knit and garden. (Focus Group 5, Participant 20)*

The most frequently reported factor negatively impacting midwives’ mental health among survey respondents was their perception that their profession was poorly understood by other health professionals (89%). Respondents were equally likely to report that their skills and experience were either valued or not valued by their healthcare professional colleagues (37%). Positive relationships with nursing staff and consultants positively impacted some respondents’ mental health (43% and 37% respectively). In contrast, 35% of respondents reported that their relationships with consultants and hospital nursing staff negatively impacted their mental health. Participants described how poor interprofessional relationships created to stress, anxiety, and feelings of lack of control. Some reported interprofessional relationships in hospitals as tense and disrespectful. As one participant explained,

*[Y].ou never kind of know what you are in for. Whether or not you are going to have a good consultant or a bad consultant or if nursing is going to be helpful and supportive or if they are going to fight you on everything. So that is hard…we are still such a small group of people and there still are doctors who think we can’t do anything. Or having to refer back to them for ordering certain things and it’s just always kind of— like we’re always fighting for validation or fighting for recognition. It never seems to end. So that I mean definitely it wears you down. Even if you have good relations with some consultants, the bad ones can really wear you down. (Interview 1)*

A corollary of midwifery being poorly understood by other professions is that midwives often feel ‘othered’ or a sense of not belonging when in the hospital setting, which negatively impacts their mental wellbeing. For example, participants described how in some hospitals midwives were excluded from secure off-site access to hospital information systems. Participants also reported other logistical or operational issues demonstrating inadequate support at the hospital level for midwives and midwifery clients who access hospital care. Overall, participants believed that midwifery was not a well-respected profession, and reported that challenges in hospitals and interprofessional disrespect significantly added to their mental health challenges.

### Preparation for practice

Some participants felt inadequately prepared for some nonclinical aspects of their job prior to entering their careers. This resulted in stress or anxiety that negatively impacted mental wellbeing. Reported gaps included information about the business aspects of midwifery, including the remuneration process. Participants felt that a business course on these aspects of their profession should have been included in their education and explained that this lack of knowledge resulted in them feeling powerless as a new registrant and ignorant about the adequacy of their compensation.

*I feel like the [undergraduate midwifery program] could really address a lot of that…we’re a business, and Business 101 is not a course…What is a caseload variable?.. How much do we get? How much does a practice get? Is it the same across practices? Who gives us this money? How do we apply for this money? As a midwife who spends many, many extra hours working with special populations, do I have access to this cash flow?.. How do you run a midwifery practice?.. A lot of my understanding is hearsay, because I am a) afraid to ask, but b) ashamed to show that I don’t know, that I should know this information. (Focus Group 2, Participant 5)*

Additionally, some participants felt that their midwifery education had not adequately prepared them for the risks of burn-out, mental illness, and culture shock as a new registrant.

*[T]he [undergraduate midwifery program] seems to paint a certain picture as to what midwifery looks like… Then, coming into practice was very different…it was just, ‘Well, this is how it is. It’s how it’s always been.’ Where there’s midwives who have lots of physical issues, and that’s just part of it. They’re unhealthy…and you’re going to have anxiety, and that’s just part of it… ‘You’ve agreed to be a midwife, and you knew what you were getting yourself into’ is what I’ve been told many a times. (Focus Group 3, Participant 11)*

Overall, findings from the survey suggest that the perceptions described by the focus group and interview participants are shared by a substantial portion of all Ontario midwives. Respondents indicated that their level of preparedness upon graduation for running a business (75%) and for non-clinical aspects of midwifery (59%) negatively impacted their mental health. 44% of respondents did not feel adequately prepared for the business and management aspects of the profession. Survey respondents reported a range of experiences related to the transition from being a student to being a new registrant, with 30% reporting a negative impact on their mental health with the transition and 41% saying that the transition had a positive impact on their mental health.

### The COVID-19 pandemic

Both phases of this study were conducted during the COVID-19 pandemic. Participants were informed that the impact of the pandemic was not the focus of our research; nonetheless, they expressed that the pandemic had impacted midwives’ mental health negatively by exacerbating existing uncertainty and stress. Participants described providing more personal support for clients when clients’ support systems were limited by public health restrictions. They also described juggling increasing workloads and new responsibilities, which negatively impacted their mental health. In contrast, some participants perceived that the pandemic was positive for interprofessional relationships as team members became more engaged with each other, and in some instances, worked together to face emerging challenges.

## Discussion

Our mixed-methods study is the first comprehensive investigation into factors that impact the mental health of Ontario midwives. Our analysis revealed that Ontario midwives’ mental health is negatively impacted by factors related to the nature of midwifery work itself, provincial midwifery funding arrangements and their implications, the culture within the profession, and the external context within which the profession exists. By drawing on a socio-ecological understanding of mental health and well-being, the inter-relationships between these factors may be understood by seeing the work experiences of individual midwives as nested within relationships within organizations within policy within society, with more micro-level factors being shaped by meso- and macro-level factors [26]. In our presentation of the results, we included descriptions of findings in which participants noted these influences. Our online survey of Ontario midwives revealed a diversity of experiences related to factors influencing mental health, but broad support for solutions such as providing more options for alternative work arrangements and part-time work for midwives, support and time off following traumatic work experiences, and access to mental health care from professionals familiar with the unique challenges of midwifery. Our findings offer insight into factors that impact midwives’ mental health in a context in which the vast majority of midwives work in a midwifery-led continuity of care model.

Our findings align with and expand upon the findings of previous Canadian and international research investigating midwives’ mental health. Previous Canadian research has highlighted the negative impacts of unpredictability and uncertainty on midwives’ mental health, associated with the on-call model [2, 20, 27, 28]. A pan-Canadian study of midwives found that unpredictable schedules and the uncertainty of being called in to work had great personal costs to the midwives, resulting in difficulties balancing work with personal lives [27]. Another study of Western Canada (British Columbia and Alberta) midwives found that 34% of midwives had considered leaving midwifery, 84.8% of whom cited the negative impact of the unpredictable on-call model [2]. Finally, a study of caseload midwives in New Zealand found that the unpredictability of caseload work had the potential to increase the level of occupational burnout [28].

Several previous studies have identified the risk of PTSD and burnout as a consequence of exposure to trauma at work for midwives [6–8]. The literature suggests that the majority of midwives will experience at least one traumatic perinatal event in their careers [8, 29, 30], which is significantly associated to burnout in midwives[6, 31] and may impact midwives’ intention to stay in the profession [8, 32]. This makes it imperative to address the impacts of trauma on midwives and ensure that midwives are aware of the potential impact of vicarious birth trauma [33], have adequate time off following traumatic experiences [20], and receive appropriate support following such experiences [32, 34], as these steps may help reduce the burden of trauma on midwives’ mental health.

Previous research has also identified the impact of interpersonal conflict, with bullying, harassment, and poor interprofessional relationships identified as a major reason for midwives leaving their practice group or the profession [2, 35, 36]. Our findings offer novel nuances to our understanding of how tensions within the profession impact mental health, including the impact of a culture within the profession of ‘midwifery machisma’ or ‘pushing through’ and the power structures that have arisen in some communities as a consequence of midwifery funding flowing through practice groups in the context of managed growth of the profession which has created monopolies. Additionally, while previous research about midwifery has shown that the public [37–42] and other healthcare providers [43–45] misunderstand the role, education, and scope of midwifery, we believe our research is the first to articulate how this lack of understanding negatively impacts midwives’ mental health. We did not apply a gender-based lens to our analysis, but it is important to note that the 2018 HRTO decision found that midwives in Ontario are subject to ongoing prejudices, stereotyping, and barriers due to gender-based discrimination, and the submission of the Association of Ontario Midwives to the HRTO included documentation of the psychological harms this had on midwives [46].

### Strengths & limitations

Our study has several strengths. Use of a mixed methods design allowed us to present rich descriptions of participants’ perceptions of factors that influence their mental health as well as to corroborate the insights of the 24 participants in Phase I of our research more broadly through a survey that was open to all midwives in the province. We used member-checking and triangulation of both data and investigators to ensure the rigor of our qualitative findings, and the response rate to our survey provided sufficient power to support the validity of our quantitative findings describing the perceptions of the profession as a whole. Triangulation of qualitative and quantitative methods was an additional strength of our mixed methods approach. Nevertheless, our findings have some limitations. First, some findings are specific to the Ontario context (e.g., funding arrangements, model of care), and therefore may not be generalizable to other settings; however, it is important to note that generalizability is not the objective of qualitative investigations. Second, participants in Phase I were more likely to have a disability or chronic illness and to be racialized than respondents in Phase II. While the proportion of Phase II respondents who self-identified as Indigenous and/or Black was very similar to the demographics of the profession in Ontario, the proportion who identified as racialized in Phase I was higher than in the provincial midwifery population. While it is important when interpreting the qualitative results to keep in mind that midwives who participated in Phase I may have been motivated to participate because of experiences that differed from the general population of Ontario midwives, we also consider it a strength that the voices of midwives who might experience marginalization were strongly represented in Phase I, and hypothesize that the leadership of the racialized members of our research team in Phase I contributed to participants feeling safe enough to accept targeted invitations to participate. Furthermore, use of the survey in Phase II allowed us to corroborate our findings based on a representative sample of Ontario midwives. Third, it was beyond the scope of this analysis to develop a comprehensive theory and explain the relationships between the factors identified in our themes. Future research might strengthen our understanding of this topic by applying gender-based or intersectional analytical lenses in developing theory.

### Implications

We propose five broad recommendations to improve the mental health of midwives based on our findings and other existing literature: (1) provide a variety of work options for midwives, (2) address the impacts of trauma on midwives, (3) make mental health services tailored for midwives accessible, (4) support healthy midwife-to-midwife relationships, and (5) support improved respect for and understanding of midwifery. Previous research has found that midwives want alternative working options, like a reduction in on-call time, different models of care, flexible working hours, and shiftwork options, and that the lack thereof contributes to their stress and burnout [2, 27, 35, 47]. Providing a variety of work options for midwives supports them to continue to utilize their specialized knowledge and skills when personal or family factors prevent them from working in a full-time, on-call, midwifery-led, continuity-of-care model. We need to ensure that midwifery students and practicing midwives are prepared for traumatic workplace events and have access to trauma-focussed intervention when indicated. Research suggests promising effects of an intervention in the UK designed to prevent PTSD in midwifery which involves an educational workshop, access to trauma-focused clinical psychology intervention, peer support, and informational leaflets [48], and other interventions aimed at improving midwives’ mental health through mindfulness and/or meditation also show promising results [49, 50]. We identified a strong desire among Ontario midwives for individual therapy and continuity of care with a mental health professional who has expertise in midwives’ mental health. Peer-support or group-based mental health support options for midwives are also recommended in the literature to cope with traumatic events and/or poor mental health.^19,20,27^ Access to tailored mental health supports for midwives can be facilitated through on-line services, but also needs to be financially accessible. Supporting healthy midwife-to-midwife relationships and improving respect for and understanding of midwifery are areas that call for novel interventions and multi-pronged approaches. Addressing both these recommendations will also require examining ways in which policies such as funding arrangements impact both intra-and inter-professional relationships and developing policy-level solutions.

## Conclusion

As the first comprehensive investigation into midwives’ mental health in Ontario, this study has identified major factors that negatively impact midwives’ mental health, including the nature of the work, the remuneration model, the culture of the profession, and external factors. To improve midwives’ mental health, we recommend providing a variety of work options for midwives, addressing the impacts of trauma on midwives, making mental health services tailored for midwives accessible, supporting healthy midwife-to-midwife relationships, and supporting improved respect and understanding of midwifery with other allied health professionals. Given that a wide range of factors contribute to poor mental health among Ontario midwives, a comprehensive response is needed to support midwives’ wellbeing. In addition to improving overall wellbeing within the profession, improving midwives’ mental health may positively impact retention of the midwifery workforce.

## Data Availability

The datasets generated during and/or analysed during the current study are not publicly available due to participant confidentiality but are available from the corresponding author on reasonable request.

## Abbreviations

PTSD: post-traumatic stress disorder
HRTO: Human Right’s Tribunal of Ontario
AOM: Association of Ontario Midwives
HiREB: Hamilton Integrated Research Ethics Board
DASS: Depression, Anxiety and Stress Scale
OB: obstetrician
EMCM: expanded models of midwifery care
HR: human resource
